# Nasopharyngeal Chordoma: A Case Report

**DOI:** 10.7759/cureus.58636

**Published:** 2024-04-20

**Authors:** Hessa Alsubaie, Rashed Aldoseri, Mohamed Alshehabi, Mai Nasser

**Affiliations:** 1 ENT, Bahrain Defence Force Hospital, Riffa, BHR; 2 ENT, Bahrain Defense Force Hospital, Manama, BHR; 3 ENT, The Royal Medical Services, Manama, BHR; 4 Otolaryngology & Head and Neck Surgery, The Royal Medical Services, Riffa, BHR

**Keywords:** primitive notochord, chordoma, ear nose throat (ent), adenoid hypertrophy, nasopharyngeal

## Abstract

Chordoma is a rare malignant neoplasm arising from remnants of primitive notochord. The most common location for chordoma is in the sacrum. This case presents a 10-year-old medically free male who came to the ENT clinic with the impression of adenoid hypertrophy. After further investigations, including imaging and biopsy, it was found to be a nasopharyngeal chordoma. Our aim, in this case, is to increase the suspension of differential diagnosis of nasopharyngeal masses other than adenoid hypertrophy. In addition, it highlights the importance of imaging in the evaluation of nasopharyngeal masses.

## Introduction

Chordoma is a rare malignant tumor that originates from the remnants of the primitive notochord in the embryo, a primitive cell line around where the skull base and the vertebral column develop. The remnants of the notochord persist in or near the midline, which is closed by bone. Chordomas are categorized as locally invasive, but they rarely metastasize [1]. However, chordoma represents 1% of intracranial tumors and 4% of all primary bone tumors. Intracranial chordomas account for 1/3 of all chordomas and occur in the vicinity of the clivus (spheno-occipital bones) [2]. In this paper, we present a case of a 10-year-old male with a history of a nasopharyngeal mass with complaints of snoring, mouth breathing, and episodes of apnea. He had the impression of adenoid hypertrophy at the beginning, and after more investigations, he was diagnosed with nasopharyngeal chordoma and received proton radiation therapy.

## Case presentation

**Figure 1:**
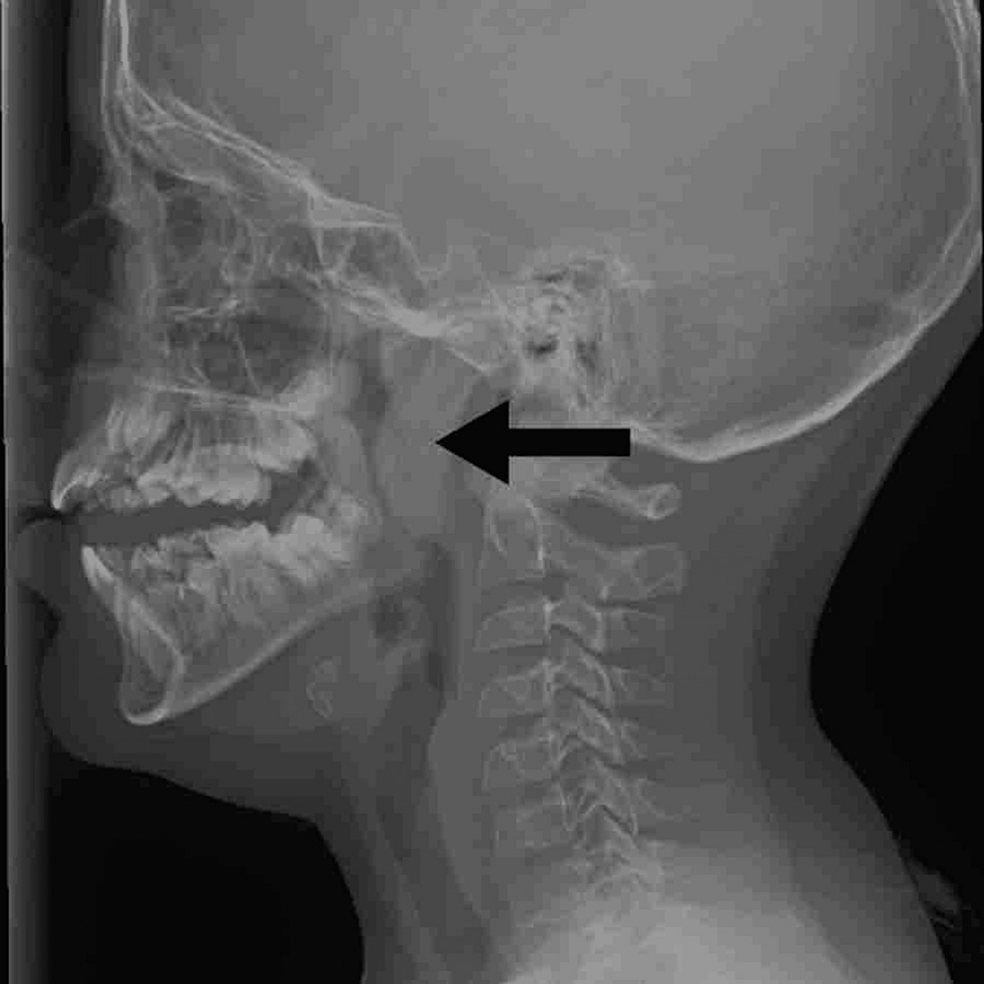
Lateral X-ray showing a nasopharyngeal filling mass Posterior nasopharyngeal soft tissue shadow encroaching on the air column is impressive for adenoid hypertrophy.

A 10-year-old medically free male presented to the ENT clinic in our hospital in 2020 with a history of snoring, nasal blockage, and mouth breathing. The patient also had some episodes of apnea that were witnessed by the mother. These complaints were there for approximately a year, and he was not on any medications. The patient denied any history of recurrent throat infections, chest infections, chronic rhinitis, chronic cough, or epistaxis. Nasal examination showed bilateral hypertrophied inferior turbinates. Throat examination showed grade 2 tonsils. Furthermore, a lateral neck X-ray showed a large adenoid (Figure 1). The plan for the management of this patient was medical treatment, including nasal steroid spray and oral anti-histamine. The patient was seen in the clinic after three months for reassessment. He reported persistent symptoms. He was still having snoring, nasal blockage, and mouth breathing despite the usage of the given medications. For that reason, the management plan changed to adenoidectomy.

**Figure 2:**
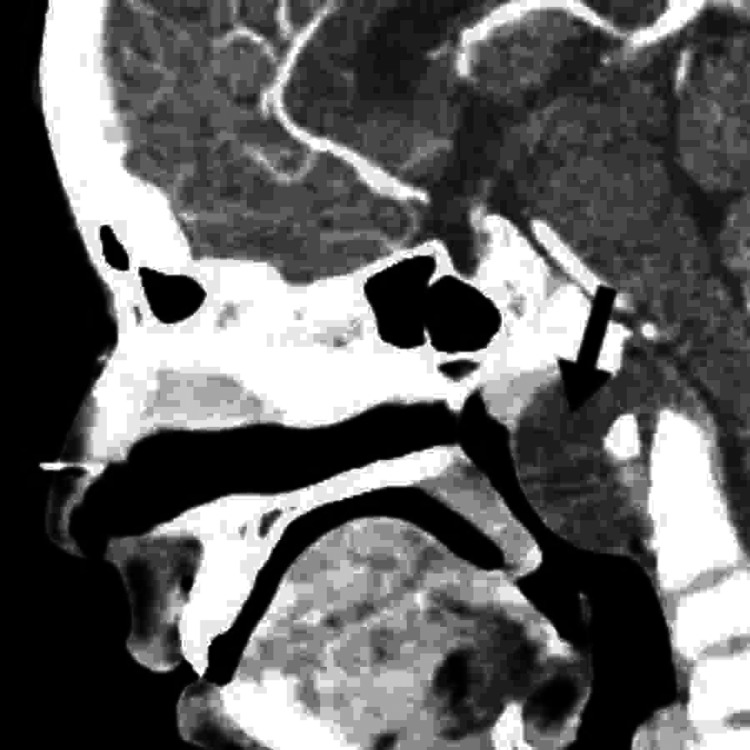
CT scan showing a defined cystic nasopharyngeal lesion A well-defined cystic lesion is in the posterior nasopharyngeal region, measuring 2.5 × 3.3 × 5 cm in dimensions.

However, CT sinuses were ordered before proceeding to the surgery to rule out other pathologies. CT sinuses reported the presence of a focal, fairly defined soft tissue lesion noted in the posterior aspect of the nasopharynx extending along the posterior parapharyngeal spaces and the anterior aspect of the foramen magnum. The lesion measures approximately 5 × 3 cm in maximum dimensions and demonstrates enhancement in the post-contrast sequences (Figure 2). MRI of the brain was also done, showing a focal, fairly defined soft tissue lesion noted in the posterior aspect of the nasopharynx extending along the posterior parapharyngeal spaces and also the anterior aspect of the foramen magnum. The radiologist gave a differential diagnosis, including vascular lesions like hemangioma or cyst. Given these radiological findings, further evaluation was needed. CT chest, abdomen, and pelvis were negative for metastasis.

**Figure 3:**
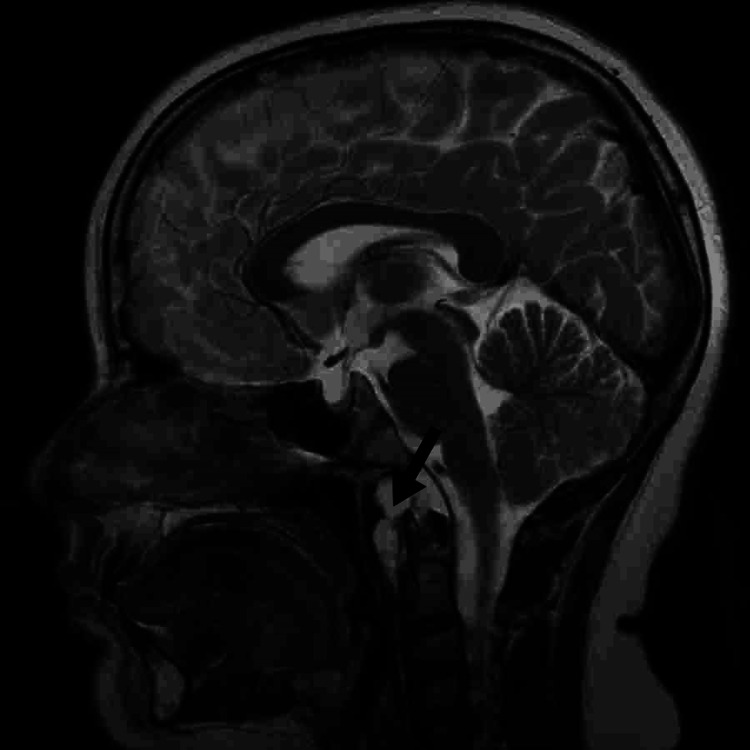
MRI showing no newly identified lesions There is no evidence of new abnormalities and no significant interval changes.

The patient was booked for endoscopic nasopharyngeal cyst excision, which was done successfully on March 20, 2021. Intraoperatively, shaving of adenoid tissue was done initially, which led to an encapsulated mass that was seen in the posterior nasopharynx area. A biopsy was taken and sent to the pathology department. The biopsy result showed fragments of tumor tissue composed of epithelioid to spindle cells forming short cords and sheets with many single large cells with abundant vacuolated cytoplasm, all within the myxoid matrix. Mild to moderate nuclear pleomorphism and occasional mitosis were also seen. In the immunohistochemistry studies, the tumor cells were positive for EMA and Pan CK. For this diagnosis, the case was discussed in the Bahrain tumor board, which advised for a proton radiation therapy in Germany. The patient was seen in Germany and advised for proton radiation therapy and refused the idea of surgical resection. Having said that, the patient had finished 38 sessions of proton radiation therapy uneventfully. The patient’s condition was stable after that, with no active complaints. A serial MRI was done, showing significantly improved local control with no recurrence until now (Figure 3).

## Discussion

Chordoma is a rare tumor that originates from the remnant of the primitive notochord in the embryo, and it is locally aggressive and develops along the cranio-spinal axis [2]. The incidental rate of chordoma is less than 0.1 per 100.00 per year, and it accounts for 1-4% of all primary bone tumors [3]. Chordoma can occur in all age groups. However, most of the cases are diagnosed during adulthood and rarely affect children and adolescents [4]. The most common location of chordoma is in the sacrum, and it accounts for 50-60% of the cases, followed by the skull base, which accounts for 25-35% (near the sphene-occipital area). The cervical vertebrae account for 10%, and thoracolumbar vertebrae account for 5% of cases [5]. 

The clinical signs and symptoms will differ depending on the anatomical location of the tumor. The symptoms may vary from compression of the vital structures, headaches, visual field defect, or obstruction if it extends into the nasopharyngeal area, as in our case [4]. However, the onset is usually asymptomatic, so it is difficult to diagnose chordoma early. For the evaluation of chordoma, CT scans and MRI imaging are usually required. Due to the involvement of the bone, a CT scan is needed, and for the involvement of the nearby structures, MRI imaging is needed. The gold standard modality that is used in the evaluation of pre- and post-treatment is MRI imaging. On a CT scan, the tumor will appear centrally located with expansile soft tissue that originates from the clivus with the association of the destruction of the lytic bone. On T2-weighted MRI, there will be high signal intensity [2,6].

Based on the locations of the mass, the differential diagnosis will differ. In our case, the differential diagnosis list is different from the differential for the classical chordoma [7]. For that reason, the differential diagnosis for our case is vascular lesions like hemangioma, cysts, and nasopharyngeal malignancies. Chordomas can be classified into three categories: classical (conventional), chondroid, and dedifferentiated. The classical (conventional) type is slow-growing and consists of physaliphorous cells within a myxoid matrix, which may show cellular proliferation. Furthermore, the chondroid type resembles a hyaline cartilage neoplastic. Moreover, the dedifferentiated type is rapidly growing and can lead to metastatic spread with the worst prognosis, and it consists of a mesenchymal component and sarcomatoid appearance. On immunohistochemistry, chordoma will show positive stains for S100 protein, vimentin, epithelial membrane antigen, and cytokeratins [4,7,8].

The primary modality in the management of chordoma is surgery. Local recurrence rates, in addition to survival rates, are shown to be dependent on the achievement of negative surgical margins, with recurrence rates on the order of 70% in cases where negative margins are not achieved [3,5]. In a series of 52 patients, Boriani et al. reported that 100% of patients treated with radiation alone, palliative therapy, or intralesional intracapsular excision had local recurrence within 17-20 months. However, only 20% of patients treated with en bloc resection with appropriate margins had local recurrence at 56-94 months [9]. The usefulness of radiotherapy as a primary or adjuvant treatment has been debated. In contrast, some investigators have reported a slight effect with radiotherapy. On the other hand, others described improved local control and prolonged disease-free survival after radiotherapy as adjuvant treatment [10].

## Conclusions

Chordoma is a rare tumor that arises from remnants of the primitive notochord. The nasopharynx area is one of the locations where chordoma may present, and it is important to consider it as one of the differential diagnoses of nasopharyngeal masses. Imaging with a CT scan and MRI, along with its pathological assessment, is needed to confirm the diagnosis. The treatment options include surgical resection and radiotherapy and follow-up with serial MRI.
